# Histopathological changes in epithelium of hair follicles and acrosyringium caused by measles in child^☆^^☆☆^

**DOI:** 10.1016/j.abd.2019.02.015

**Published:** 2020-02-12

**Authors:** Monique Freire Santana, Luiz Carlos de Lima Ferreira, Joao Gabriel Nogueira de Oliveira, Fábio Francesconi

**Affiliations:** a Department of Teaching and Research, Fundação Centro de Controle de Oncologia do Amazonas, Manaus, AM, Brazil; b Pathological Anatomy Management, Fundação de Medicina Tropical Dr. Heitor Vieira Dourado, Manaus, AM, Brazil; c Dermatology Medical Residency Program, Fundação de Medicina Tropical Dr. Heitor Vieira Dourado, Manaus, AM, Brazil; d Universidade Federal do Amazonas, Manaus, AM, Brazil

**Keywords:** Giant cells, Hair follicle, Measles

## Abstract

Some epidermal alterations in measles has been described, such as keratinocytes apoptotic, parakeratosis, giant-cell formation, intranuclear and cytoplasmic inclusions, dyskeratosis, spongiosis, and intracellular edema. The authors report for the first time in human a case of measles with the presence of multinucleated giant cells in the hair follicle and dyskeratosis in acrosyringium.

A 9-year-old boy evolved with headache and fever. Seven days after the onset of symptoms, coughing, an episode of hemoptysis, coryza, and conjunctivitis, followed by a rash that started on the forehead with a cephalophaudal progression. He had no comorbidities or family history.

The exanthema was distributed in a cephalocaudal direction, with appearance of pruritic maculopapular lesions, some confluent plaques, more intense on the face and trunk. The physical examination revealed a hypochromic macula, with diffuse erythematosus halo, before the appearance of a rash on the oral mucosa, Koplik's sign (Fig. 1), besides erythematous papules and conjunctival hyperemia. The patient had not completed the measles vaccination scheme. A biopsy was performed in the dorsal region three days after the onset of the rash, in order to corroborate to the clinical diagnosis.

**Figure 1:**
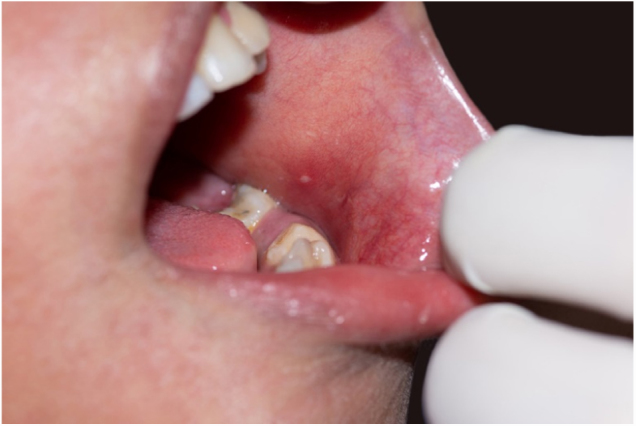
Koplick's sign: whitish papule surrounded by erythema.

## Histopathologic findings

**Figure 2:**
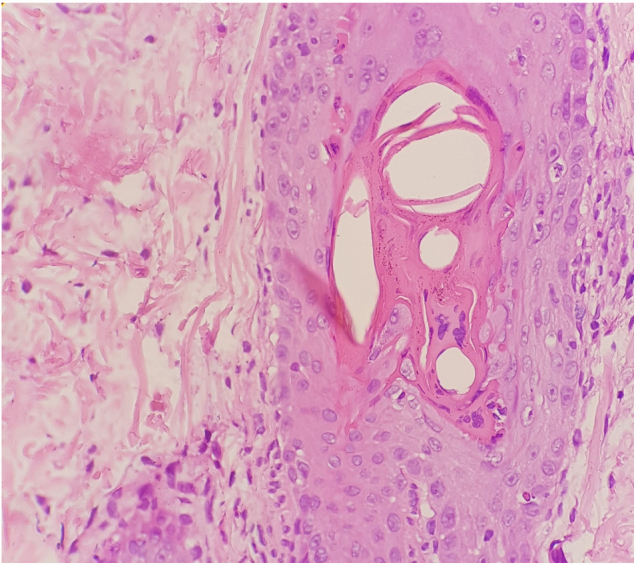
Dyskeratosis and multinucleated giant cells in follicular epithelia (Hematoxylin & eosin, ×400).

**Figure 3:**
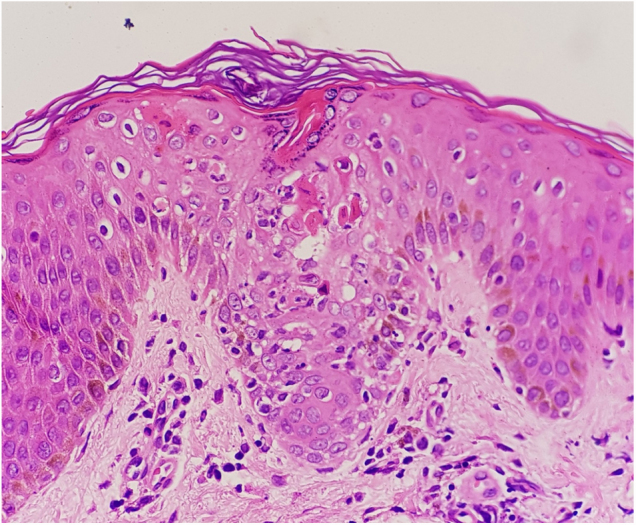
Dyskeratosis and mild spongiosis in acrosyringium (Hematoxylin & eosin, ×400).

The skin biopsy showed hyperkeratosis, mild spongiosis, dyskeratosis, focal parakeratosis, and apoptotic keratinocytes. In follicular epithelia, was observed dyskeratosis and multinucleated giant cells (Fig. 2). The dyskeratotic alterations also compromised the acrosyringium (Fig. 3).

## Discussion

Torres, in 1952,^1^ describes in a case study the histopathologic changes in a skin biopsy of measles according to the time of biopsy. Ten hours after the eruption, the changes restricted to the epidermis, with spongiosis and karyolysis. Hyaline necrosis of epidermal cells either singly or in clumps represents the primary change in the measles eruption. In lesions biopsied after 12 h, Torres described the formation of small vesicles with necrotic keratinocytes and a few polymorphonuclear cells. At this time, he observed multinucleate epithelial giant cells in the stratum spinosum. Perivascular infiltration by mononuclear cells occurs 12 h after the eruption. In the dermis, it was described edema of the papillary layer and perivascular infiltrations in the reticular layer by large mononuclear cells some of them containing small irregular deeply stained granule, so-called Mallory-Medlar-Lipschütz cells also described.

The most common findings are parakeratosis, giant-cell formation, intranuclear and cytoplasmic inclusions, dyskeratosis, spongiosis, and intracellular edema.^1^, ^2^ It can also be described as an inflammatory infiltrate of mononuclear cells with eosinophils and capillaries with fibrin thrombi.^3^ Electronic microscopy examination can show capsid particles, within the endoplasmic reticulum and secretory vesicles measuring approximately 40–60 nm in diameter,^3^ and aggregates of microtubules within the nuclei and the cytoplasm of syncytial giant cells in both epidermal and oral epithelial lesions.^2^

Ewing^4^ described some findings at the hair follicles in measles, such as hydropic vacuoles in the epidermis; edema, and an increase of large round cells of dermis; edema, and various degrees of degeneration of cells of the hair follicle. The vesicle formations occur in connection with similar changes in the sebaceous glands and hair follicles, being nearly always extensively affected.

Epithelial giant cells in the hair follicles were described, in our knowledge, only by Hall et al.^5^ in skin biopsies from Rhesus monkeys with measles rash, without documentation in humans. To the best of our knowledge, this is the first report that describes the giant cells of measles in hair follicles and dyskeratotic alterations in the acrosyringium.

## Financial support

None declared.

## Authors’ contributions

Monique Freire Santana: Approval of the final version of the manuscript; conception and planning of the study; elaboration and writing of the manuscript; obtaining, analysis, and interpretation of the data; critical review of the literature; critical review of the manuscript.

Luiz Carlos de Lima Ferreira: Approval of the final version of the manuscript; conception and planning of the study; effective participation in research orientation; critical review of the manuscript.

João Gabriel Nogueira de Oliveira: Elaboration and writing of the manuscript; obtaining, analysis, and interpretation of the data.

Fábio Francesconi: Effective participation in research orientation; intellectual participation in the propaedeutic and/or therapeutic conduct of the studied cases; critical review of the manuscript.

## Conflicts of interest

None declared.
